# Hedgehog Morphogens Act as Growth Factors Critical to Pre- and Postnatal Cardiac Development and Maturation: How Primary Cilia Mediate Their Signal Transduction

**DOI:** 10.3390/cells11121879

**Published:** 2022-06-09

**Authors:** Lindsey A. Fitzsimons, Victoria L. Brewer, Kerry L. Tucker

**Affiliations:** 1 Graduate School of Biomedical Science and Engineering, University of Maine, Orono, ME 04469, USA; 2 Department of Biomedical Sciences, College of Osteopathic Medicine, University of New England, Biddeford, ME 04005, USA; vbrewer@une.edu

**Keywords:** Sonic Hedgehog, Indian Hedgehog, primary cilia, organogenesis, congenital heart disease, heart development

## Abstract

Primary cilia are crucial for normal cardiac organogenesis via the formation of cyto-architectural, anatomical, and physiological boundaries in the developing heart and outflow tract. These tiny, plasma membrane-bound organelles function in a sensory-integrative capacity, interpreting both the intra- and extra-cellular environments and directing changes in gene expression responses to promote, prevent, and modify cellular proliferation and differentiation. One distinct feature of this organelle is its involvement in the propagation of a variety of signaling cascades, most notably, the Hedgehog cascade. Three ligands, Sonic, Indian, and Desert hedgehog, function as growth factors that are most commonly dependent on the presence of intact primary cilia, where the Hedgehog receptors Patched-1 and Smoothened localize directly within or at the base of the ciliary axoneme. Hedgehog signaling functions to mediate many cell behaviors that are critical for normal embryonic tissue/organ development. However, inappropriate activation and/or upregulation of Hedgehog signaling in postnatal and adult tissue is known to initiate oncogenesis, as well as the pathogenesis of other diseases. The focus of this review is to provide an overview describing the role of Hedgehog signaling and its dependence upon the primary cilium in the cell types that are most essential for mammalian heart development. We outline the breadth of developmental defects and the consequential pathologies resulting from inappropriate changes to Hedgehog signaling, as it pertains to congenital heart disease and general cardiac pathophysiology.

## 1. Introduction

**Figure 1:**
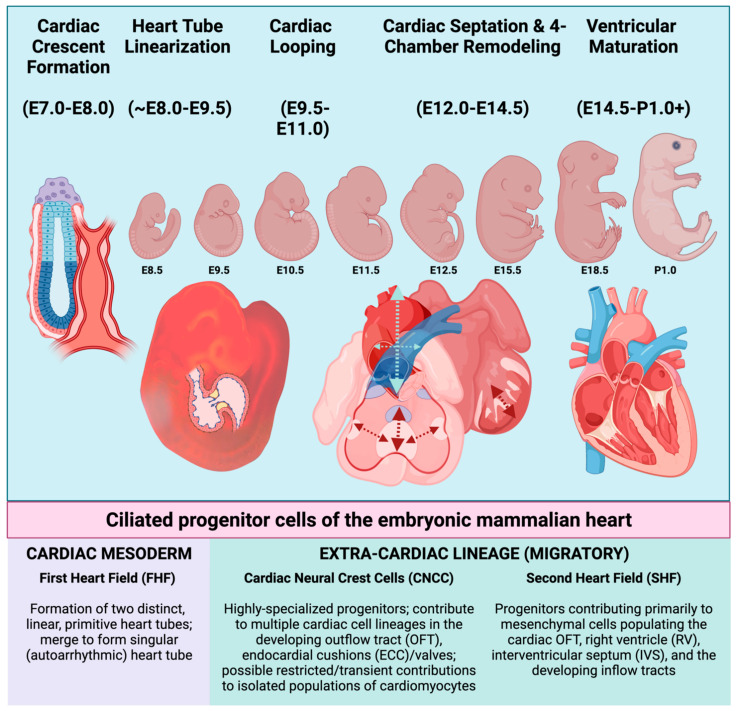
Schematic overview of embryonic heart development in the mouse. Embryonic days of gestation (“E”) are indicated beneath each stage and indicate the number of days post-coitus. Mammalian cardiogenesis involves an intricate number of developmental stages, many of which are orchestrated by, and are dependent upon, the presence of intact primary cilia (From left to right). Broadly speaking, embryonic heart development in the mouse can be divided into stages involving the initial formation of cardiac crescent/primitive heart tube, linearization of the heart tube, cardiac looping (to orient the future chambers in space), cardiac septation, and ventricular remodeling and maturation. Each of these stages requires cellular contributions from one or more of the progenitor cell populations indicated beneath the illustration. These progenitors elaborate primary cilia and originate either intrinsically from within the cardiac mesoderm, or they must migrate to the developing heart from an extra-cardiac location. Upon arrival near or within the developing heart, CNCC and SHF progenitors will later differentiate into many of the terminally differentiated cell types. First heart field (FHF); cardiac neural crest (CNCC); outflow tract (OFT); endocardial cushions (ECC); second heart field (SHF), right ventricle (RV); and interventricular septum (IVS). Figure created with Biorender.com (accessed on 29 May 2022).

Embryonic heart development in the vertebrate involves a highly complex, tightly coordinated series of developmental signaling and gene expression patterning (Figure 1), many of which rely upon the primary cilium [1,2,3]. Here, we present a summary model of murine and human embryonic cardiac development, followed by an introduction to the primary cilium structure and the sensory functions that are most relevant to the developing mammalian heart.

**Figure 2:**
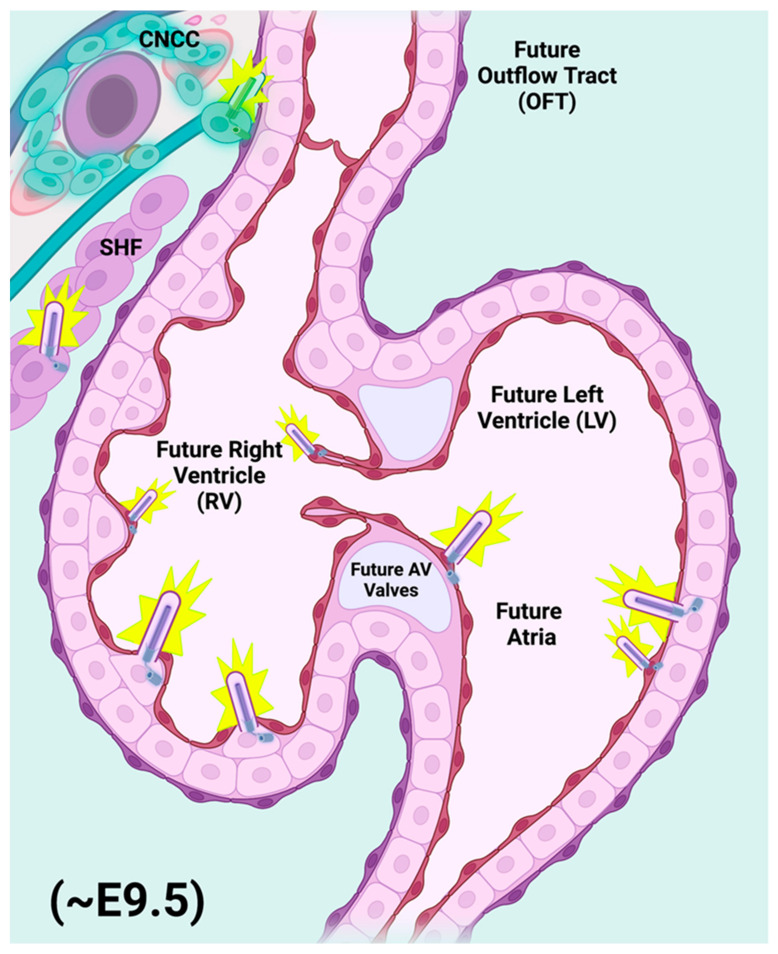
Primary cilia populate nearly all cell types of the developing embryonic heart. Representative E9.5 mouse heart showing elaboration of primary cilia (purple rods) from the cells of the migratory cardiac neural crest (CNCC) and second heart field (SHF) populations. As the embryonic heart begins to formalize its relative size and orientation, it receives an influx of migratory progenitor contributions from both CNCC and SHF, which contribute both directly and indirectly to the developing cardiac outflow tract (OFT), endocardial cushions (ECC), and ventricular myocardial components during the final stages of cardiac looping and the beginnings of cardiac maturation phases, primarily septation of the OFT, and of the atrial and ventricular chambers. Primary cilia are also elaborated from many of the differentiated cells found within the heart chambers, including endothelial/endocardial and myocardial cells. The presence of a primary cilium on any one of the above cell types (progenitors included) inherently renders that cell with the sensitivity to respond to and even to be dependent upon Hedgehog signaling for appropriate proliferation, laterality, orientation, polarity, and cell survival cues that are critical to mammalian heart development. The size and relative distribution of primary cilia illustrated above not to scale. Figure created with Biorender.com (accessed on 29 May 2022).

Embryonic mammalian heart development can be broken down into distinct, transitional phases: Establishment of the cardiac crescent (mouse embryonic day (E) E7.0–E8.0, i.e., 7.0–8.0 days post coitus); formation and linearization of the heart tube (E8.25–E9.5); cardiac looping (E9.5–E11.0); four-chamber remodeling (E12.5–E14.5); and ventricular maturation (E14.5–perinatal day 1.0). Early blastocyst formation and subsequent gastrulation leads to the establishment of three germ layers of embryonic tissue: the outer ectoderm layer, the middle mesoderm layer (including the hematopoietic, atrial, and ventricular cardiac mesoderm), and the inner endoderm layer [4]. Formation of the cardiac crescent by mouse embryonic day E8.0 is brought about by the contributions of progenitor cells from a more mature, pharyngeal endoderm, and two rapidly proliferating cardiac progenitor populations, the first heart field (FHF) and the second heart field (SHF). The FHF is the first to establish an organized cardiac structure(s) that is referred to as the primitive heart tubes, which form bilaterally from the cardiac crescent and then merge to form a singular, elongated heart tube by ~E8.0. Contributions from the SHF assist with the early formation of the cardiac outflow and inflow tracts, which are visible by ~E8.0 [5]. The linearization of the heart tube refers to the merging/elongation of the singular cardiac tube, which results in the alignment of the cardiac tube to form the developing cardiac outflow tract (OFT), early right and left ventricles, and primitive right and left atrial chambers by ~E8.5. At this critical stage (~E9.0–E9.25), the developing heart begins to receive contributions from a highly specialized, migratory group of cardiac progenitor cells known as cardiac neural crest cells (CNCC). CNCC are a subset of neural crest cells that originate from the dorsal aspect of the developing neural tube (Figure 2). CNCC migrate ventrally toward the developing heart, reaching their primary destination of the cardiac OFT by ~E9.5 (Figure 2). Along with additional contributions from the SHF to the OFT and the developing ventricular chambers, the looped heart then undergoes a serious of cellular expansions (proliferation) in an elegantly orchestrated phase of cardiac development that is referred to as the four-chambered septation phase [6]. As indicated by name, the septation phase is the phase of cardiac development whereby the previously patent heart tube becomes physically separated into the four distinct cardiac chambers that are characteristic of the adult mammalian heart: the right and left atrial and ventricular chambers that will eventually separate pulmonary from systemic circulation during the early postnatal period. The arguably most arduous stage of cardiac development occurs in the final stages of gestation, where the muscular, ventricular chambers of the heart undergo a maturation process known as ventricular compaction. Thought to be primarily conducted via tightly regulated communication between the developing endocardium and the ventricular myocardium lying beneath it, the compaction of the ventricular myocardium is a dynamic and still relatively mysterious morphogenetic process. The end product of compaction is a reduction in the thickness of the inner/spongy trabecular myocardium, and the expansion/thickening of the outer, compact ventricular myocardium. The compaction process is of critical relevance, as this maturation of the ventricular myocardium is a rate-limiting step that permits the organization and formation of both the coronary vasculature, as well as the ventricular portions of the cardiac conduction system.

**Figure 3:**
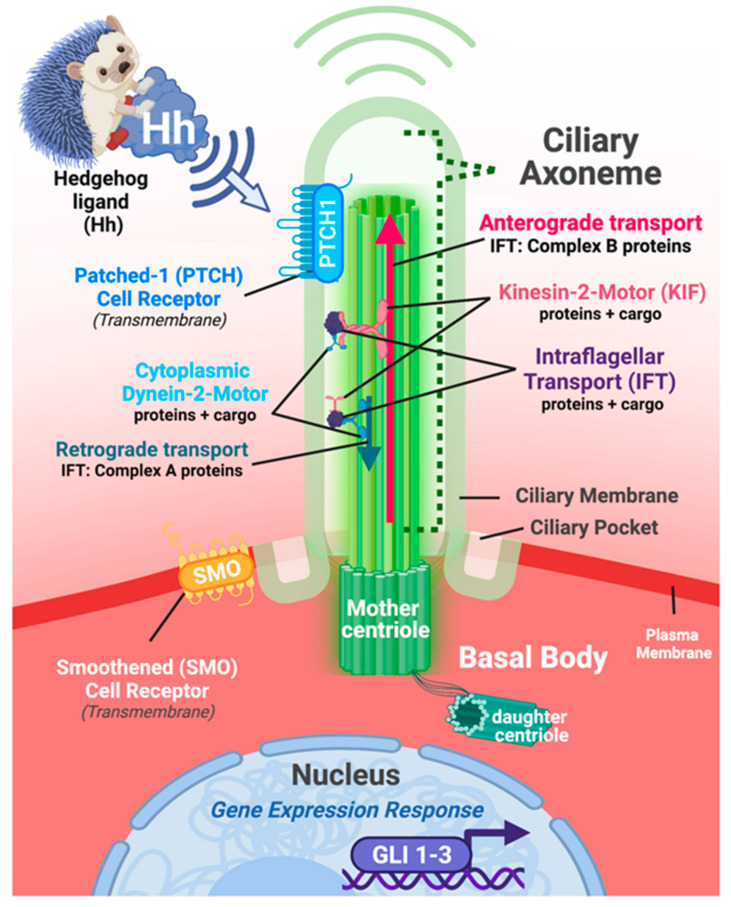
Primary cilia are dynamic, sensory organelles tasked with interpreting changes to and within the intra- and extracellular environments. Illustrative representation of the approximate composition and location of a generic eukaryotic cell and its intact primary cilium. On the apical surface of the cell, the primary cilium forms its axonemal portion as an extension of the plasma membrane protruding outward towards the extracellular environment. The ciliary axoneme is structurally supported within by the 9 + 0 microtubule scaffold that anchors the cilium to the body of the cell via the basal body complex, which is composed of both mother and daughter centrioles. Experimental modeling of cells, tissues, and diseases involving damage to or loss of primary cilia typically involve the genetic manipulation of genes and proteins critical for maintaining intraflagellar transport (IFT; protein + cargo) and/or kinesin-2-motor protein transport (protein + cargo). Elaboration of the cilium and maintenance of protein transport in the anterograde direction requires IFT Complex B proteins. Conversely, retrograde transport requires IFT Complex A proteins. In both directions, IFT proteins are acting as a scaffold to connect the respective motor complexes to the transported cargo components. Many receptors localize to the plasma membrane/ciliary membrane of the axoneme, the ciliary pockets and/or basal body region of the primary cilium complex, and can subsequently be affected, should genetic and/or environmental damage to the cilium occurs. The Hedgehog signaling pathway is closely associated with the primary cilium and serves as a critical regulator of mammalian development. Transduction of canonical Hedgehog signaling involves two co-receptors, Patched-1 (Ptch1) and Smoothed (SMO), both of which shuttle dynamically between the plasma membrane portion of ciliary axoneme itself and just outside of the base of the cilium. Hedgehog signal transduction leads to modification in the expression and protein activity of GLI gene family members encoding DNA-binding transcription factors (GLI 1-3). Size, length, and general structure of the cell/primary cilium illustrated above reflect a generalized example for illustrative purposes; Figure created with Biorender.com (accessed on 29 May 2022).

Primary cilia are antenna-like structures that protrude from almost all eukaryotic cells types. They range in size from 1 to 3 μm in endothelial cells, and up to 9 μm in neural cells (Figure 3) [7]. These organelles are known to function in the propagation and regulation of key sensory functions, both inter- and intracellularly in both normal and pathogenic environments [8]. The central core of the primary cilium comprises an axoneme of nine microtubule doublets arranged around a central space devoid of microtubules [9]. This sui generis “9 + 0” transverse pattern is the characteristic of the primary cilium, distinguishing them from motile cilia, which contain two central microtubules and associated appendages in the central space [10]. The microtubule doublet central core is employed for a process known as intraflagellar transport (IFT) that is responsible for bidirectional trafficking of proteins into and out of the cilium, and thus, is not only essential for the assembly of the structure of the growing cilium, but also maintains the axonemal structure [11]. IFT in mammals utilizes KIF3A, a kinesin-II motor protein complex driving anterograde transport. In contrast, cytoplasmic dynein enables retrograde transport. IFT in both directions utilizes “IFTXX” proteins, in which the symbol “XX” reflects their respective mass in kilodaltons. IFT88, for example, is a protein of 88 kilodaltons mass. Interaction between the protein cargo and the two motor systems is mediated by these IFT scaffolding proteins, divided into classes A and B, for retro- and anterograde transport, respectively [11]. Early analysis of *Kif3a* or *Ift88* knockouts in the mouse has shown a necessity for the presence of primary cilia in developing mice [12,13]. The deletion of the IFT complex or motor proteins results in the depletion of primary cilia. This ciliary depletion is detrimental to development, and leads to ciliopathies in humans [14].

The process of ciliogenesis of the primary cilium in mammalian cells can vary, but generally begins during the G_0_ (quiescence) or G_1_ (cell growth) phases of the cell cycle, and elongation of the primary ciliary axoneme continues through the S stage (DNA synthesis), or until the duplication of the centrosome components of the basal body have completed. Prior to its entry into the M phase (mitosis), the cilium is resorbed [15]. The anchoring of the primary cilia axoneme to the cell membrane is initiated at and maintained by the basal body complex, which is composed of both mother and daughter centrioles. After cells enter G_0_ or G_1_, the centrioles migrate to the plasma membrane where the mother centriole docks and forms the basal body substrate used to elaborate the ciliary axoneme [10]. Prior to cell division, the cilium must be disassembled. Disassembly of the primary cilium involves multiple biological processes, and at least in certain cell cultures systems, it occurs primarily via a rapid “shedding” mechanism of the axoneme, together with its plasma membrane sheath, though a gradual resorption of the cilium back into the cellular cytoplasm also occurs [16].

Although primary cilia were first described well over 100 years ago [17], it was not until much later on that scientists began to suspect that this organelle’s function spanned far beyond a vestigial nature [18,19]. Not only is this nonmotile, plasma membrane extension inherently unique in its ultrastructure, but the primary cilium is also incredibly dynamic, functioning within and outside of eukaryotic cells in multiple, pathogenically, and clinically significant ways [19]. Dysfunction occurring to prevent, limit, or later destroy these processes have been shown to impair overall sensory/signaling function and has been associated with a number of pathological disease conditions, known as ciliopathies [20]. Ciliopathies are commonly referred to as a spectrum of diseases, because of the fact that primary cilia have been implicated in various pathological processes ranging from congenital fibrocystic disease of the developing organs [21] to acquired and age-related diseases such as obesity [22], osteoporosis [23], and a growing number of cancers [24,25,26]. Examples of ciliopathies include asphyxiating thoracic dysplasia, Leber congenital amaurosis, nephronophthisis, polycystic kidney disease, and Alström, Bardet–Biedl, Ellis-van Creveld, Joubert, Kartagener, McKusick–Kafman, Meckel–Gruber, orofaciodigital, Senior–Løken, and Sensenbrenner syndromes [14] 

In the following sections, we describe the morphological, functional, and signaling mechanisms that are most relevant to furthering our understanding of primary cilia and their contributions to the pathogenesis of congenital heart defects (CHD). Specifically, we emphasize the importance of Hedgehog signaling throughout key developmental time points that are critical to embryonic heart development.

## 2. Primary Cilia and Their Function in the Development of Specific Cardiac Structures 

### 2.1. Primary Cilia within the Heart: Distribution and Phenotypic Consequences Attendant with Ciliary Dysfunction

Starting in the mid-1960s, primary cilia were first observed in embryonic and adult hearts from various organisms, including rabbits, mice, and lizards, using transmission electron microscopy (TEM) [1,27]. This microscopic approach revealed the “9 + 0” microtubule doublet organization that is characteristic of the primary cilium (Figure 3). Originally, TEM was employed as a gold standard to unequivocally identify the organelle and distinguish it from other cilia-like plasma membrane protrusions such as motile cilia, stereocilia, and microvilli. Currently, immunofluorescence techniques employing antibodies that specifically recognize axonemal components of the cilium are generally presented in research articles as a simpler and quicker approach to identify cilia. By employing scanning electron microscopy, Van der Heiden et al., demonstrated that monocilia are present on endocardial cells in embryonic chicken, with a lower frequency on endothelial cells [28]. The primary cilia were oriented strikingly, projecting into the blood-filled chambers of both the ventricles and atria. Subsequent immunohistofluorescence analysis revealed the presence of primary cilia in all three layers of the embryonic heart in mid-gestation mouse embryos: in the endocardium, the myocardium, and even the epicardium [29,30], as summarized in Figure 2. Primary cilia were found within the mesenchymal cells of the endocardial cushions [29,31,32]. These cilia showed a random orientation within the cushion, whereas endocardial cells oriented their cilia to the lumen of the outflow and inflow tracts. TEM analysis showed that these cilia bore the identifying “9 + 0” orientation of primary cilia, whereas no cilia with the “9 + 2” orientation characteristic of motile cilia were observed [29,31,32]. Slough et al., have demonstrated that cilia are found in the mouse embryo heart as early as E9.5 [32]. Interestingly, *Kif3a*−/− mouse embryos lacking cilia showed defective development of the endocardial cushions and compact myocardium at E9.5. The authors have also investigated the consequences of the genetic manipulation of nodal cilia in the mouse embryo, where structurally normal but immobile cilia result in mutants with an abnormal left–right cardiac asymmetry (*Ird* and *PKD2* mutants) [32]. These embryos demonstrated abnormal development of endocardial cushions but possessed an apparently normal myocardium.

Primary cilia have been shown to have a role as fluid shear stress sensors of kidney epithelial cells in culture [33], although the exact nature and extent to which this mechanism functions is of ongoing debate [3,34,35]. A correlative study compared the distribution of primary cilia on endothelial and endocardial cells and the chicken embryonic expression pattern of the high shear stress marker Krueppel-like factor-2 [28]. They revealed an inverse correspondence between the expression of this marker and the distribution of primary cilia. In areas where Krueppel- like factor-2 is not detected, primary cilia are present, and the shear stress is expected to be low. Even though not all of the endothelial and endocardial cells have a primary cilium, they all are shear stress-responsive. The authors propose that the cytoskeleton can function as a transducer of shear stress, and that the primary cilia in the endothelial and endocardial cells sense changes in shear stress. The low-shear forces are transmitted to the cytoskeleton, and a shear stress response is generated; these shear stresses could, in principle, contribute to the morphological elaboration of the chambers in the heart [28]. Indeed, the loss of primary cilia has been shown to change the propensity of the endothelium-to-mesenchyme transition that is critical for the formation of the endocardial cushions [36].

Defects in heart development in cilia mutants were actually recorded well before cilia were known to play a role in development. Mutations in the polycystin-2 gene (*PKD2*) lead to autosomal dominant polycystic kidney disease [37]. *PKD2* encodes an integral membrane glycoprotein that shares similarities with calcium channel subunits, and is localized to primary cilia. Wu et al., generated a knock-out of *PKD2* and mice homozygous for the mutation demonstrated a failure to form the interventricular septum and, to a lesser extent, the interatrial septum [38]. Boulter et al., described mice carrying a targeted mutation in the polycystin-1 gene *PKD1*, which, like *PKD2*, encodes an integral membrane protein that both localizes to the primary cilium and interacts with *PKD1*. Again, heart defects, including hemorrhagic pericardial effusion, atrio-ventricular septal defects, disorganization and thinning of the myocardial wall, and double-outlet right ventricles were observed [39]. Willaredt et al., examined a hypomorphic allele of *Ift88* and made the first comprehensive overview of the consequences of global primary cilium loss for the middle-third period of embryogenesis [31]. They reported many common CHD phenotypes, including persistent truncus arteriosus (PTA), and atrial (ASD) and ventricular (VSD) septal defects. An examination of a complete, targeted deletion of *Ift88* revealed defects in the OFT and reduced ventricular trabeculation [40].

### 2.2. Primary Cilia and Hedgehog Signaling: A Long-Standing History

Although the complexities of the developing mammalian heart require multiple combinations of local and circulating growth factors at multiple cell type- and cell fate-specific timepoints, Sonic Hedgehog (SHH) continues to be one of the primary mitogens that are critical to multiple aspects of mammalian heart development. Genetic and functional aberrancies in Hedgehog pathway genes independent from and along with protein expression profiles have been closely linked with nearly all anatomical manifestations of CHD, including OFT defects, atrio-ventricular septal defects, cardiac arrhythmia, cardiac hyperplasia, and multiple cardiomyopathies [41,42]. SHH functions as a unique growth factor, in the sense that the biological effects of the ligand’s activity are not only contextually dependent, but are also highly sensitive to deviation from normal gradients [43]. Both their overexpression/upregulation as well as the under-expression/downregulation of the Hedgehog signaling pathway components lead to the formation of developmental defects and/or the pathological remodeling of postnatal tissue. Hedgehog thresholds must be acutely maintained in a highly tissue- and temporally specific manner. A failure to appropriately suppress Hh thresholds (i.e., too much Hedgehog) as seen in scenarios of Hedgehog overexpression and gain-of-function experiments, leads to the formation of CHD. A failure to initiate or sustain adequate Hh thresholds (i.e., too little Hedgehog), as seen in loss-of-function experiments, genetic knockout, siRNA knockdown, and Hedgehog antagonists, also results in CHD. Thus, building a healthy heart requires a precise titration of Hedgehog signaling to meet the near-instantaneous cellular and tissue demands of embryonic tissue. 

The Hedgehog gene/signaling pathway was first discovered in *Drosophila melanogaster* 45 years ago, where fruit fly embryos with mutations in the *Hedgehog* gene failed to develop correctly, lacking wings and properly organized denticles [44]. The disorganized and bunched-up denticles closely resembled the morphology of the spikes on a hedgehog, and the Hedgehog gene family was eventually determined to be an important mediator of genes involved in the tissue patterning, polarity, and laterality of nearly all metazoan embryos [44]. Subsequent discoveries revealed three Hedgehog genes in mice and humans, including *Shh*, Desert Hedgehog (*Dhh*), and Indian Hedgehog (*Ihh*) homologues, while there is only one known Hedgehog ligand in invertebrates [45]. In mammals, SHH controls, among a plethora of organ systems and embryonic processes that for lack of space we cannot enumerate, left–right asymmetry and the development of the lung and heart [46,47,48,49]. DHH is an important element in spermatogenesis, and it also has a role in the development of the perineural sheath of peripheral nerves [45,50]. IHH mainly functions in the growth and differentiation of chondrocytes, and in the development of the endochondral skeleton [12,51,52], although it is also involved at the embryonic node [47].

**Figure 4:**
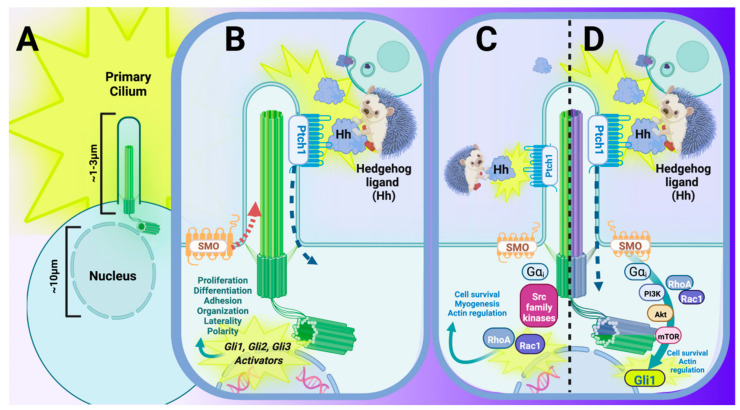
The primary cilium and its interaction with Hedgehog (Hh) signal transduction is critical to many aspects of the developing mammal and maintenance of postnatal mammalian tissue. (**A**) Embryonic primary cilia extend approximately 1–3 μm into the extracellular environment and function as a highly specialized sensory organelle that interpret changes in the surrounding environment in nearly all mammalian cell types. (**B**–**D**) The Hh signaling pathway is the pathway that is most closely associated with and dependent upon an intact primary cilium in mammals. (**B**) Canonical Hh signaling involves the secretion of Hh ligand from a Hh-secreting cell (upper right panel), which is bound by Patched-1 (PTCH1), a 12-pass transmembrane receptor. Binding of the Hh ligand to PTCH1 initiates a conformational change that leads to translocation of the Hh–Ptch1 complex just outside of the ciliary axoneme while also permitting its 7-pass transmembrane co-receptor, Smoothened (SMO), to translocate within the ciliary axoneme. Permissive relocation of SMO into the primary cilium concurrently allows GLI activators to enter into the nucleus for transcriptional activation and/or further upregulation of Hh signaling. (**C**,**D**) Noncanonical Hh signaling also utilizes Hh ligand secreted from a Hh-secreting cell (upper right panel). Noncanonical pathways for Hh signaling are best-characterized by their functions independent from either a traditional Hh receptor (C, Type II Noncanonical), Hh functioning independently from one (or all) of the GLI transcription factors (**C**), or a Smoothened-independent, non-traditional route of activating GLI targets (D, Type I Noncanonical). (**C**) One example of noncanonical Hh signal transduction can occur in the presence of Hh ligand, where activation of the pathway is achieved via SMO, and the subsequent initiation of RhoA/Rac1-mediated mechanisms of proliferation, cell adhesion, and/or migration. (**D**) Another example of noncanonical Hh is brought about in the presence of Hh ligand, where binding to PTCH1 activates a SMO-independent, G-protein coupled receptor-like activation of PI3K (via Gαi), and the subsequent initiation of GLI1 transcriptional activation. Figure created with Biorender.com (accessed on 29 May 2022).

Like many growth factors that are critical to development, the SHH signal cascade can be categorized broadly into canonical and noncanonical mechanisms (Figure 4). Canonical SHH signaling involves the secretion of the ligand from one cell, and the activation of the Hedgehog signaling cascade when SHH binds to one of its receptors, Patched-1 (PTCH1), which is located on the plasma membrane of the primary cilium on an adjacent or nearby cell [53]. Upon binding/activation, PTCH1 translocates to just outside the base of the primary cilium, permitting its co-receptor, Smoothened (SMO), to translocate into the primary cilium [54], where a downstream family of glioma-associated (GLI) transcription factors acts to further activate or to repress a variety of biological processes, including proliferation, differentiation, polarity/organization, and migration within the cell itself, and/or of neighboring cells/tissues [55,56]. Canonical Hedgehog signaling requires the primary cilium, as well as activation of GLI transcription factors, where SMO functions as one readout of the activity of this pathway [47]. Of note, GLI1 is understood to function almost exclusively as an activator for Hedgehog signaling, and it can be interpreted independently or along with increases in *Ptch1* expression as a readout(s) for the activation/upregulation of the Hedgehog signaling pathway [57].

The Hedgehog co-receptor, SMO, is classified as a member of the Frizzled family of G-protein coupled receptors (GPCRs; Class F) [58]. The SMO protein contains several components characteristic of GPCRs, including seven transmembrane domains, an amino-terminal cysteine rich domain, three intra- and extra-cellular loops, and an intracellular carboxyl-terminal tail. These features enable SMO GPCR signaling, with an expanding understanding of regulatory contributions from alternate functional domains beyond the post-translational modification potentials of the SMO carboxyl tail.

Noncanonical Hedgehog signaling, by contrast, describes the activation of Hedgehog signaling without the requirement for all of the canonical molecular players [59,60], and importantly, also without the necessity of primary cilium involvement. Noncanonical signaling can be further broken down into Type 1 and Type 2 subtypes (Figure 4C,D). Type I noncanonical Hedgehog signaling (Figure 4C) is described as the activation of the Hedgehog/GLI pathway, dependent upon ligand binding to the PTCH1 receptor, but mediated by the novel functions of PTCH1, potentially via the integral membrane proteins CAM-related/downregulated by oncogenes (CDO), brother of CDO (BOC), and/or Growth arrest-specific-1 (GAS1) [61,62,63,64], and independent of SMO involvement in the transduction pathway. This particular route of Hedgehog activation is relevant, given that BOC, CDO, and GAS1 have been shown to promote cardiogenesis in pluripotent stem cells [61] (Figure 4C), and may also play co-mediator roles in the regulation of the pathways critical to apoptosis, CREB/ERK/GPCR, and general promoters of myogenesis and axon guidance [62,63,64,65]. Type 2 noncanonical Hedgehog signaling is dependent on the Hedgehog ligand and on SMO, but not on the activation of all traditional GLI transcription factors to carry out a specific biological response (Figure 4D). Rather, Hedgehog ligand binding to PTCH1 activates inherent functional GPCR properties of SMO, whereby the activation of heterotrimeric G_i_ proteins are responsible for the downstream activation of such proteins as phosphoinositide 3-kinase (PI3K), Ras homologous family member A (RHOA), and Ras-related C3 botulinum toxin substrate 1 (RAC1) [66] (Figure 4D). While noncanonical Hedgehog signaling is less critical during embryogenesis, this is often the signaling cascade that is involved in disease initiation and progression via the reactivation of developmental regulatory pathways [65,67]. In addition, upregulation of the Hedgehog signaling pathway and activation of GLI transcription factors independent of either SMO activation, and even of the ligand itself, has been previously identified as one of the primary drivers of disease [65,67].

The data discussed in the following sections indicate that cardiac cilia and the associated Hedgehog signal cascades are clearly essential for normal heart development. The individual sections will focus on specific components of the embryonic heart in which primary cilia have been shown to be important. However, many of the developmental and compensatory mechanisms that are involved in the observed cardiac defects remain poorly understood. 

### 2.3. Cilia of the Embryonic Node

The embryonic or ventral node (called Henson’s node in chickens and Kupffer’s vesicle in zebrafish) is a spherical cluster of progenitors cells that merges with the primitive streak during late gastrulation, and is visible by E7.5 in mouse embryos [68]. The arrangement of cilia at the node is unique, in that the primary cilia are present surrounding the nodal ring, but the inside of the space is occupied by nodal cilia. Nodal cilia are specialized embryonic monocilia (one per cell) that are important in directing the laterality of the organs of the thorax and abdomen [48]. They are similar to primary cilia, in that they lack a central microtubule doublet within the axoneme. By contrast, nodal cilia ultrastructure is characterized by the presence of inner and outer axonemal dynein arms that are located in between microtubules doublet pairs. These dynein arms serve to connect one microtubule doublet to the adjacent/preceding microtubule doublet in a manner that results in the bending motion observed in nodal and motile cilia [69]. This unique ultrastructure allows nodal cilia to move in an active swirling motion to mediate left–right patterning during late gastrulation and prior to the onset of organogenesis [48]. Dysfunctions in nodal cilia can lead to profound disturbances in heart development in both human and mouse models. In order to focus on the later events directed exclusively by primary cilia, we refer the reader to two excellent recent reviews on the downstream consequences of nodal dysfunction for heart development [2,3]. 

### 2.4. Primary Cilia of the Migratory CNCC Population

In order to investigate the mechanisms of the CNCC cilia and CNCC-mediated CHD, Willaredt et al., used a hypomorphic allele of the *Ift88* gene entitled *cobblestone* (*cbbs*), generated by an ENU mutagenesis in mice [31]. In the *cbbs* allele, the expression of a normal IFT88 protein is reduced to 25% of wild type levels, thus producing a model where cilia are globally reduced in the developing mouse heart [31]. An examination of CNCC migration in the absence of primary cilia was conducted using antibodies recognizing the plasma membrane-spanning low-affinity pan-neurotrophin receptor p75, which is expressed by migratory neural crest cells [70]. This analysis revealed that p75-positive migratory CNCC cells were unaffected by the loss of primary cilia, and were able to populate within the developing pharyngeal arches and cardiac OFT in the *cbbs*^−/−^ mutant animals. By contrast, the elaboration of primary cilia was required for the subsequent septation and maturation of the developing heart (OFT), where *cbbs*^−/−^ mutants displayed a severe CHD phenotype, including the formation of a single OFT (PTA), atrioventricular septal defects (AVSD), enlarged pericardial sac, dilated atrial chambers, and embryonic lethality by ~E13.5 [31]. Interestingly, a diverse array of great vessel defects was revealed, including transposition of the great vessels, double aortic arch, and *arteria lusoria*. The examination of *Shh* expression revealed no changes in homozygous mutants, but both the *Gli1* and *Ptch1* Hedgehog-pathway readouts were substantially reduced in the pharyngeal mesenchyme of the mutants, indicating a reduction in Hedgehog signaling [31].

### 2.5. Primary Cilia of the Endocardial Cushions

The developing heart valves are referred to as endocardial cushions, because of their sac-like appearance within the developing heart. Over 20 years ago, it was reported that mitral valve regurgitation and prolapse are common in patients with polycystic kidney disease type I [71]. More recent findings have suggested a key role of primary cilia in the development of the mitral valve. Firstly, mutations in the cadherin protein DCHS1 (Dachsous homolog gene 1) has been shown to cause mitral valve prolapse in humans [72]. DCHS1 is an atypical member of the cadherin superfamily that has recently and surprisingly been localized to the membrane domain found at the base of motile cilia elaborated by adult human respiratory epithelia [73]. Its widespread expression during embryogenesis in tissues that bear primary, but not motile, cilia, argue that it may also function specifically at the primary cilium. Secondly, mutations in the Filamin-A gene cause familial cardiac valvular dystrophy [74]. Filamin A is an actin-filament binding protein that can localize to the basal body, and it works with the transmembrane protein MKS3 (Meckelin), which functions in centriole migration to the apical membrane, to promote ciliogenesis [75]. Thirdly, a series of elegant studies have examined the DZIP1 (DAZ Interacting Zinc Finger Protein 1) protein, which has a dual role in the regulation of Hedgehog signaling [76]. DZIP1 interacts with GLI3 and prevents it from entering the nucleus, while it also interacts with IFT88 and CEP164, a centriole-localized protein, to promote ciliogenesis [76]. Specifically, it has been found that a loss of primary cilia during murine embryonic development has led to mitral valve leaflet enlargement, characterized by an expansion of the extracellular matrix and histological disruption, present at birth [29]. This led to an adult myxomatous valve pathology that is comparable to that seen in mitral valve prolapse [29]. Crucially, the authors employed both a conditional knock-out of *Ift88*, as well as a mouse mutant in the *Dzip1* gene, bearing a DZIP1 mutation seen in patients with mitral valve prolapse, thereby showing a brilliant complementary usage of the two species to elucidate CHDs at the translational level. Interestingly, the Hedgehog ligand controlling this process is DHH, marking a distinct exception to the rule that SHH is controlling most of the processes in cardiac development [77]. The presence of cilia with regard to valve development has been connected to the type of extracellular matrix that was produced, and it has been well-characterized by Toomer and colleagues [29,78]. Cilia were specifically found most abundantly where there were proteoglycan-rich regions of extracellular matrix, suggesting a role or connection to the composition of the extracellular matrix during various stages of development [29]. There is also evidence that the presence of cilia on the heart valves is important during embryonic development, but that the cilia disappear after birth. This work by Toomer et al. [29], in addition to that of Li and colleagues [42], have further connected ciliopathies identified in murine hearts to human genes. 

The most common human birth defect, and thus also the most common CHD, is a bicuspid aortic valve (BAV) in roughly 1–3% of births. This condition has been associated once before with Joubert syndrome, one of the best studied ciliopathies [79]. Primary cilia have been found to be present on the interstitial cells of developing OFT cushions at E11.5 and E13.5, and also on the valve endocardium [78]. *Ift88* conditional knockout mice were found to display a BAV phenotype with a 68% penetrance, indicating that primary cilia on the aortic valves are both present within and play a crucial role in aortic valve morphogenesis [78]. The authors demonstrated a unique spatiotemporal arrangement of cilia on the valves, consistent with what has been previously described as the presence of cilia in areas of low-grade shear stress [78]. Interestingly, a later study using genome-wide association protocols in BAV patients revealed single-nucleotide polymorphisms in genes that regulate ciliogenesis through the exocyst, which bring the ciliary cargo to the plasma membrane. In humans, defects in this process leads to both BAV as well as valvular stenosis and calcification [80]. Doubtless, mouse models of these mutations will allow for a clear mechanistic explanation of the link between exocyst function and the downstream signal cascades leading to BAV.

### 2.6. Primary Cilia of the Atrio-Ventricular Septa

#### 2.6.1. Atrial Septation

Hofmann et al., have kept track of the location of Hedgehog-responding cells, using an R26R^Gli1−CreER2^ mouse model induced by a single dose of tamoxifen at E7.5 [81]. Hedgehog-responsive cells were identified using beta-galactosidase to mark Cre-positive recombination [81]. Their data show a migration of the Hedgehog-receiving cardiac progenitors from the posterior SHF splanchnic mesoderm into the atrial septum between embryonic ages E9.5 and E11.5 [81]. Further observations in the cardiac OFT have revealed that, also between the ages of E9.5 and E11.5, migration of Hedgehog-receiving cardiac progenitors occurs from the pharyngeal mesoderm into the pulmonary artery. Mice with a conditional allele of *Smo* were then used in order to investigate the importance of Hedgehog signaling in atrial septation. Deletion of *Smo* in Hedgehog-responding heart progenitors with a tamoxifen-inducible Gli1::Cre transgene revealed that all mutant embryos showed atrioventricular septal defects. These results demonstrate that proper cardiac septation is dependent upon the ability of cardiac progenitors to respond to Hedgehog guidance. These results are consistent with the phenotype observed in both the *Shh* knock-out mouse [82] and *Ift88* hypomorph ENU mutant [31]. Taken together, it is clear that regardless of how Hedgehog loss-of-function is achieved, cardiac progenitors of the atrial septum require both primary cilia and Hedgehog signaling to properly septate the atrial chambers of the developing heart.

#### 2.6.2. Ventricular Septation

Ventricular septation defects are not only the most common CHD, but one of the most common congenital defects known to our species. VSDs have been recorded in over two dozen cilia mutants in the mouse and human [13], and we will focus only on two examples here. Similar to the method used to generate the *cbbs* allele of *Ift88* [31], an ENU mutagenesis screen was carried out in the mouse to identify novel mutations in the atrioventricular septation process. One mutant line ensuing from the screen (*Dnah11^avc4^* allele) was determined to bear a missense mutation in the gene encoding Dynein Axonemal Heavy Chain 11 (*Dnah11*), which is actually required for motile ciliary function, as DNAH11 localizes to and enhances the integrity of the outer dynein arm [83]. Indeed, they could record no *Dnah11* expression in the embryonic SHF or heart tube [83]. The authors observed that the AVSD phenotype only occurred in *Dnah11^avc4^* mutant embryos suffering from heterotaxy. This phenotype is usually attributed to dysfunctions of the embryonic node, and the authors attribute the AVSD as a downstream consequence of perturbations in laterality determination, a general principle that has been concluded by other researchers [69,83,84]. In contrast, in the same screen, the authors identified a mutation in the primary cilium IFT gene *Ift172* and also a nonsense mutation in the *Mks1* (Meckel syndrome, type 1) gene [83]. *Mks1* expression, as expected, was identified in the SHF, and mutant embryos had decreased Hedgehog signaling. This suggests that both motile and primary cilia have independent, spatiotemporally-distinct functions in proper atrioventricular septal development. 

*Fantom* (*Ftm*, *Rpgrip1l*, or *Retinitis Pigmentosa GTPase Regulator Interacting Protein 1 Like*) is a mouse gene encoding a protein with multiple different protein–protein interaction domains. RPGRIP1L has been shown to localize to the basal body of primary cilia [85]. Investigations into homozygous *Ftm* mutant mice showed ventricular septal defects, as well as thinner ventricular septal myocardium, but no changes to the interatrial septum [86]. It was found that cilia length was significantly reduced in the embryonic ventricles of mutant mice, which correlated with a decrease in proliferation. A ventricle-specific downregulation of *Ptch1* expression was recorded, and reduction in the PDGFR-alpha signaling pathway was also identified. The investigators also noted that *Ftm* genetic mutations in humans have been observed in various ciliopathies, including patients with Meckel–Gruber syndrome, Joubert syndrome, and nephronophthisis [86]. These data suggest that cardiac primary cilia have a role in ventricular septum development that is mediated through Hedgehog signaling. Neither study indicates which key aspect of the morphogenetic changes leading to ventricular septation are fundamentally altered by the observed reductions in primary cilium function, nor whether Hedgehog signaling is the exclusive perturbation leading to VSDs.

### 2.7. Primary Cilia of Cardiac Fibrocytes

Primary cilia have been shown to play a key role in fibrosis of the heart [87], but the exact nature of this process and the exact cell types involved necessitates additional confirmation. While primary cilia are present in the ventricles of fetal murine hearts as early as 9.5 embryonic days and as late as 15.5 embryonic days, there is evidence to suggest that the observed primary cilia elaborations do not necessarily correspond to the cardiomyocytes of the ventricular myocardium. Villalobos et al., exposed adult, infantile, and neonatal rodent hearts to myocardial injury, in order to further examine how the collective ventricular myocardial cells/tissues would respond to ischemic injury [87]. A 45 min bout of experimentally induced ischemia was followed by subsequent reperfusion of the cardiac muscle. Results revealed a transient increase in ciliated, vimentin-positive cardiac fibroblasts when compared to the control group, where cardiac tissue was not exposed to ischemia-reperfusion injury. When 7-day-old mice were given an apical resection surgery, investigators observed an increase in ciliated cells 7 days post-operation. Further investigations into the origin/lineage of these cells revealed that these increases in ciliated cells did not correspond to cardiac troponin-I-positive cells (cardiomyocytes); rather, they stained positive for vimentin, a standard marker for cardiac fibroblasts [87]. Similar results were observed when repeating immunofluorescence analysis on adult murine hearts post-myocardial infarction. The authors concluded that the increases in ciliation did, in fact, correspond to increases in ciliated fibroblasts present in the proliferative healing phase [87]. Investigators also examined various molecular/structural components of individual primary cilia of cardiac fibroblasts following ischemia–reperfusion injury in the rat heart. The results of these experiments supported a working hypothesis that PC1, PC2, and KIF3A proteins are localizing to the neonatal rat cardiac fibroblasts. There was also evidence to suggest that the cilium-localized proteins PC1 and KIF3A are required for TGFβ-1 (Transforming Growth Factor Beta-1)-induced collagen biosynthesis, showing a specific functional role for the primary cilia [87]. Collectively, these results support a diverse role for primary cilia, not only in the context of developing myocardial tissue, but also in early post-embryonic and adult post-natal myocardial molecular responses/adaptations to wound repair of the ventricle.

### 2.8. Primary Cilia of the Endocardium

Primary cilia have vital roles in heart development, including allowing different areas of the embryonic heart to communicate and coordinate appropriately. It has been established that the signaling interactions between the endocardium (and the myocardium in later development) contribute to the proper development of the heart valves and the trabeculation of the ventricles [88]. Endocardial cells are defined as endothelial cells that are specifically localized within all four chamber walls of the heart [89]. While these cells do express generic markers that are consistent with endothelial specificity, including the plasma membrane-anchored cell differentiation marker CD31 (PECAM-1), they also express proteins that make them uniquely identifiable as endocardial cells; for example, the extracellular matrix glycoprotein fibronectin and the transcription complex protein Nuclear Factor of Activated T-cells 1 (NFATc1) [90].

Endocardial cells send regulating signals to the myocardium that affect their growth and maturation [91]. Different signaling pathways, including the bone morphogenic (BMP) pathway, are involved in this communication. Moreover, many of these pathways are directly dependent on primary cilia for proper functioning [31]. Another example is the endocardial-specific Notch ligand (NOTCH1B), which requires the presence of an intact primary cilium to activate the downstream expression of EPHRINB2A and NEUREGULIN-1, which are key regulators of trabeculation in zebrafish [92,93]. NEUREGULIN-1 ligand is then secreted from the ventricular endocardium, where it binds to ErbB2/4 coreceptors, expressed by the layer of cardiomyocytes beneath the endocardium, to initiate protein synthesis of cardiomyocyte and sarcomeric proteins, as well as mediating the cardiomyocyte proliferative response that is critical for the development, compaction, and maturation of the ventricular myocardium in all mammals [94].

### 2.9. Primary Cilia of the Ventricular Myocardium

Primary cilia have been documented and visualized in nearly all eukaryotic cell types [95]. The elaboration of primary cilia from terminally differentiated, adult ventricular cardiomyocytes, on the other hand, is an occurrence that remains controversial [87]. Primary cilia of murine, embryonic atrial and ventricular cardiomyocytes have been relatively well-documented with fluorescence microscopy and immunofluorescence analysis, using primary antibodies against primary cilia-specific proteins against ARL13B, IFT88, and acetylated alpha-tubulin [30,96]. However, the appearance and number of primary cilia displayed by cells within the ventricular myocardium appears to shift dramatically in the early postnatal period [30,87]. Specifically, there seems to be a pronounced loss in the ciliation of ventricular myocardial cells during the first and second postnatal weeks, after which, primary cilia appear to remain elusive until (or if) an ischemic, hypoxic, and/or undo mechanical stress stimulus is introduced to the adult/, diseased, and/or aging heart [30,87].

The capacity of the primary cilium to function as a mechanosensory organelle is well-documented in the articular cartilage of bone [51], kidneys [33,34,97], and as sensors of sheer stress in arterial vasculature [98]. Thus, consideration for a mechanosensory role of the primary cilium in ventricular cardiomyocytes and their neighboring cell types is a concept that seems not only feasible, but likely [99]. In the embryonic mammalian heart, primary cilia have been observed in the ventricular cardiomyocytes of both rat and mouse model organisms [30,100]. Primary cilia have also been documented in the cardiomyocytes of the developing zebrafish heart [93]. Whether adult human cardiomyocytes elaborate cilia remains a topic of disagreement in the field. Investigations looking into the relevance and receptiveness of the embryonic ventricular myocardium to mechanical stimuli has been previously investigated in the chick [101,102], mouse [102,103], and zebrafish [93,102] embryonic model organisms. Collectively, these studies have provided ample evidence, across multiple species, supporting the importance of laminar blood flow in maintaining the developing embryonic heart within stable, but appropriate, biomechanical ranges. Furthermore, these studies support a significant role for the primary cilium itself in sensing and interpreting mechanical stimuli (i.e., blood flow) to help shape the healthy and maturing ventricular myocardium [32,104,105]. 

At present, it is known that human embryonic stem-cell derived cardiomyocytes elaborate primary cilia in vitro [106], and that human postnatal cardiomyocytes in individuals with existing cardiovascular disease can display primary cilia [106,107]. However, formally one needs to distinguish the *ability* of an adult cardiomyocyte to elaborate a primary cilium from its *usage* of this cilium in the sensory functions that are unique to this organelle, and this should be considered in future investigations. Recent work by Villalobos et al. [87] provided a thorough analysis of primary cilia present in the ventricular myocardium, where the authors observed the presence of cilia in the neonatal and adult heart. However, further analysis led the authors to conclude that primary cilia elaboration originated from cardiac fibroblasts, and not from true, cardiac troponin-positive, expressing cardiomyocytes. 

## 3. The Multiple Roles That Hedgehog Signaling Plays in Cardiac Development and Function 

### 3.1. Hedgehog Signaling and Progenitor Cells of Mammalian Heart Development

SHH serves multiple influential but also critical functions in the developing mammalian heart that can be categorized further into extracardiac and intracardiac mechanisms of action [108]. Early expression of *Shh* that is most relevant to heart development is seen during early somite formation at E8.0. By E9.5, *Shh* expression can be seen along the dorsal aspect of the head/future spine. At this stage, *Shh* expression extend anteriorly along the ventral midline and within the prechordal plate mesoderm (pharyngeal mesoderm) [109]. From an axial perspective, *Shh* expression can be seen primarily along the floor plate of the murine notochord, just dorsal to the cardiac region of the mammalian embryo [109]. The production and secretion of SHH ligand from this region then expands, concentrating within the pharyngeal arches (and specifically, the endodermal portion) and subsequently extending a SHH gradient that spans from the pharyngeal endodermal region to the more the ventral aspect of the cardiac region of the developing embryo [43,109]. Following the formation and elongation of the primitive heart tube, SHH ligand is produced and secreted from the pharyngeal endoderm of the branchial arches 3, 4, and 6. From the extensive studies of SHH and its influence on the neural progenitors [108], we know that the SHH produced in this region are strategically positioned to help guide the migratory path, proliferation, polarity, and differentiation of the CNCC on their way to populate the OFT of the developing heart [110].

CHD is a known consequence of the deviation of the pharyngeal SHH gradient from the norm or when the ability of the migratory CNCC to respond to SHH is damaged (e.g., via the downregulation of primary cilium function) [31,111,112]. Smoak et al., provided an early characterization of the cardiac phenotype resulting from the conditional elimination of *Shh* from mouse CNCC using the tg(P0-Cre)1Ky Cre driver, which drives recombination in migratory neural crest progenitor cells [82]. Given the temporal aspects of this Cre driver, investigators were able to focus primarily on early OFT formation (E8.5–E10.5). The authors demonstrated that at this earlier stage, Shh did not act directly on CNCC progenitors, and it specifically acted to restrict the expansion of neural crest populations to those of the cardiovascular and craniofacial domains [82]. This phenomenon contrasts with what we know about the potential of SHH to act directly on differentiated CNCC, in order to promote cell survival and proliferation during the later stages of heart and OFT development (≥E10.5–E12.5) [82]. In a subsequent series of experiments, Goddeeris et al., confirmed that SHH produced in the pharyngeal endoderm was required to direct CNCC to survive and proliferate within the endocardial cushions of the developing OFT [111]. More specifically, the conditional elimination of mouse *Shh* from Nkx2.5+ cells of the secondary heart field not only led to the loss of *Shh* mRNA and SHH protein expression from the pharyngeal endoderm, but also subsequently led to the failure of the OFT to septate, resulting in the formation of PTA [111].

Concurrently with CNCC, cellular influxes from the secondary heart field (SHF) begin to populate the dorsal and inferior aspects of the embryonic heart, including the OFT and right ventricular chamber, as early as E10.5 [113]. In these complimentary and similarly migratory populations of progenitor cells, SHH that is produced by both the pharyngeal endoderm and possibly the pharyngeal mesoderm, functions to maintain an appropriate balance of proliferation [114,115,116]. Specifically, SHF cells that are present within the OFT and right ventricle differentiate into cardiomyocyte vascular smooth muscle cells that require SHH to direct appropriate levels of proliferation during the expansion, elongation, and septation of both the OFT and ventricular portions of the heart [117]. Goddeeris et al., demonstrated that by conditionally eliminating *Shh* from Nkx2.5/Cre+ (SHF)-expressing cells in the mouse, there was a noticeable disruption to endocardial cushion formation, as well as the subsequent ability of these cells to communicate with cardiomyocytes derived from the first heart field [111]. Taken together with subsequent experiments, investigators concluded that the SHH produced internally within the dorsal mesocardium, as well as the SHH produced in the dorsal mesenchymal protrusions (“extracardiac”) were both required for cellular processes and communications that are necessary for OFT and atrial formation and septation [111,116,118].

One key regulator of both SHH and progenitor cell contributions in the developing heart is the low-density lipoprotein receptor-related protein 2 (LRP2), a multi-ligand receptor that is highly expressed throughout the developing OFT in a specialized niche of cardiac progenitor cells in SHF [115]. Under normal circumstances, LRP2 expression in these SHF cells promotes sustained progenitor cell fate, which is required for the complete septation of the cardiac OFT. In *Lrp2*-deficient mouse embryo experiments, the loss of LRP2 led to the premature differentiation of SHH-dependent progenitor cells into cardiomyocytes within the OFT. Loss of Hedgehog sensitivity in these cells, combined with inappropriate differentiation, led to the shortening of the OFT, which developed fully into a common arterial trunk CHD in mutant hearts [115].

### 3.2. Hedgehog Signaling in Pre- and Postnatal Cardiomyocytes

Despite current debates regarding the presence, role, and distribution of primary cilia found in the ventricular myocardium, an expanding body of literature supports a role for SHH in the proliferation and tissue homeostasis of both pre- and postnatal ventricular myocardial tissue. From the early stages of heart development, short- and long-range Hedgehog signaling gradients are required for the proper migration and differentiation of cardiac progenitor cells, including the maintenance of progenitor status and appropriate proliferative responses of cardiac neural crest and second heart field cell populations [61,114]. Although the CNCC contribution to the ventricular myocardium is accepted in the zebrafish model organism [119,120], it is widely debated whether this phenomenon is consistent across mammalian species [87,103,121]. SHF contributions to the ventricular myocardium have long since been confirmed [111,122]. Later in cardiac development, Hedgehog signaling has been identified as being a primary regulator of both atrial and ventricular septation processes, both of which must be fully completed in order to isolate future right-heart (pulmonary) from future left-heart (systemic) circulation networks [118]. 

Hedgehog signaling-based mediation of the electrophysiology of postnatal cardiomyocytes has also been documented, where the upregulation of Hedgehog signaling via SMO can reduce potassium sensitivity and function in cardiomyocytes [123]. Decreased potassium sensitivity can lead to a prolonging of the cardiac action potential duration, subsequently predisposing ventricular cardiomyocytes to life-threatening forms of ventricular arrhythmias [123]. In addition to Hedgehog effects on cardiomyocyte-selective ion transport, the hypothesis can be formulated that Hedgehog signaling changes during heart development may affect the integrity and organization of gap-junction proteins (e.g., Connexin-40 (Cx40) and Connexin-43 (Cx43)), which are critical for the propagation of cardiac action potentials within the distal, ventricular portions of the cardiac conduction system. Although the majority of evidence to support this hypothesis has primarily been documented in chondrocytes/bone [23] and in neural tissue [124] outside of the heart, the influence of Hedgehog signaling pathways on the formation, patterning, and integrity of Connexin gap-junction proteins in the cells and tissues most relevant to cardiac function continue to support the feasibility of this hypothesis [125,126,127].

In addition to the Hedgehog influence on cardiac electrophysiological structures and function, there continues to be a growing interest in potential roles for Hedgehog in the pathogenesis and/or remediation of ischemic cardiac injury [128]. Paulis et al., demonstrated that the upregulation of the SHH pathway via multiple pharmacological interventions prior to induced ischemia–reperfusion injury resulted in a pronounced reduction in the size and arrhythmogenic capacity of the resulting cardiac infarct [129]. The authors concluded that supplementation with SHH ligand prior to this model of ischemic injury, via an increase in cardiomyocyte potassium sensitivity, could prophylactically prevent or reduce the likelihood and severity of ventricular repolarization defects [129]. Another study by Kawagishi and colleagues found that the activation and upregulation of SHH ligand expression regulated the cardiac regenerative response to the neonatal mouse heart in response to apical resection performed on the first postnatal day [130]. Furthermore, these authors provided a comprehensive characterization of SHH ligand production within the myocardium. Although the SHH ligand was determined to be produced and secreted by non-myocyte cells, this study provided convincing evidence to support the regulation of cardiomyocyte proliferation, as well as influencing the inflammatory immune response to injury via the recruitment of monocytes and macrophages to the site of damaged tissue [130]. We propose that the Hedgehog signaling-mediated phenomena described here could be dependent upon primary cilia elaborated by postnatal, adult myocardial cells. Doubtless, experiments are underway in which the primary cilia are genetically ablated from mature myocardial tissue to avoid the predictable complications that could arise if cilia are removed during the development of the myocardium.

## 4. Conclusions

Hedgehog ligands function as potent and effective morphogens/growth factors for healthy heart development. Signaling by primary cilia and Hedgehog both function as independent and co-dependent mediators of mammalian heart development. However, because of the dynamic nature of this growth factor, Hedgehog gradients and signaling thresholds must be carefully and temporally regulated at the level of the primary cilium. The regulation of Hedgehog thresholds involves consideration for the short- and long-range acting capacities of the ligand, the resilience nature/preservation of this pathway, and also the type of pathway that is utilized (i.e., canonical vs. noncanonical). Because primary cilia and Hedgehog signaling play such a critical role in the development and maturation of the mammalian heart, the potential capacity for the activation and repression of Hedgehog signaling and the primary cilia of different cardiac cell populations may offer novel mechanistic insights into cardiac congenital and acquired disease pathogenesis, as well as the potential for innovative therapeutic targets in the detection, treatment, and prevention of both CHD and acquired cardiovascular disease.

## Data Availability

Not applicable.
